# Machine actionable metadata models

**DOI:** 10.1038/s41597-022-01707-6

**Published:** 2022-09-30

**Authors:** Dominique Batista, Alejandra Gonzalez-Beltran, Susanna-Assunta Sansone, Philippe Rocca-Serra

**Affiliations:** 1 Oxford e-Research Centre, Department of Engineering Science, University of Oxford, Oxford, UK; 2 Present Address: Scientific Computing Department, Rutherford Appleton Laboratory, Science and Technology Facilities Council, Didcot, UK

**Keywords:** Standards, Data processing

## Abstract

Community-developed minimum information checklists are designed to drive the rich and consistent reporting of metadata, underpinning the reproducibility and reuse of the data. These reporting guidelines, however, are usually in the form of narratives intended for human consumption. Modular and reusable machine-readable versions are also needed. Firstly, to provide the necessary quantitative and verifiable measures of the degree to which the metadata descriptors meet these community requirements, a requirement of the FAIR Principles. Secondly, to encourage the creation of standards-driven templates for metadata authoring, especially when describing complex experiments that require multiple reporting guidelines to be used in combination or extended. We present new functionalities to support the creation and improvements of machine-readable models. We apply the approach to an exemplar set of reporting guidelines in Life Science and discuss the challenges. Our work, targeted to developers of standards and those familiar with standards, promotes the concept of compositional metadata elements and encourages the creation of community-standards which are modular and interoperable from the onset.

## Introduction

The publication and release of data, along with associated laboratory and computational methods, holds the potential of providing new research insights through meta-analysis and justifies establishing domain specific data repositories. Data that are routinely made available in a transparent and persistent manner can effectively drive science forward by enabling the necessary scrutiny of the peer-review process but also enable data science through data reuse process. This is especially true in the era of agent-driven knowledge discovery from data. However, besides the various factors hampering data sharing and which have been analysed elsewhere^1^, considerable efforts are still required to discover, harvest, clean and harmonise datasets to build data corpora of suitable quality for consumption by software and learning systems. Central to these operations is the availability of machine-readable metadata, i.e. descriptive data about the data, which provides the contextual information essential to interpret and reuse the data. In the Life Sciences, for example, descriptors of the experimental steps (e.g., provenance of study materials, measurement and technology types) and molecular entities of interest (e.g., metabolites, proteins) are essential information to ensure efficient and meaningful data reuse as well as, in principle, allow work to be reproducible. Over the years, many domain specific metadata models have been produced but disappointingly, few interoperate well.

### Metadata, a pillar of FAIR

Along with unique and persistent identifiers, metadata is the cornerstone of the FAIR Principles, guiding scientific data management and stewardship^2^. The principles promote the Findability, Accessibility, Interoperability, and Reusability of digital assets such as datasets, algorithms and models, emphasising the need for machine-readability of the data. Widely endorsed by communities in the academic and private sectors^3^, as well as infrastructure providers, scholarly publishers, funders and other global organizations^4,5^, FAIR has quickly become a fundamental enabler of digital transformation.

Currently, providing guidance on how to produce richly described metadata (metadata authoring), and how to evaluate digital object FAIRness levels^6^ (FAIR assessment) at scale represent two major challenges to implementing FAIR effectively and meaningfully. For tools and automated systems to assist with metadata authoring and compliance assessment, we need canonical metadata profiles of these reporting standards against which to create or measure the level of annotation compliance. These profiles should be “readable” and “actionable”. The latter form indicates a shift in maturity status^7^, which allows a software agent to exploit the formal representation and understand its content, rather than just obtaining a string without any context, as it occurs in a read action.

**Fig. 1:**
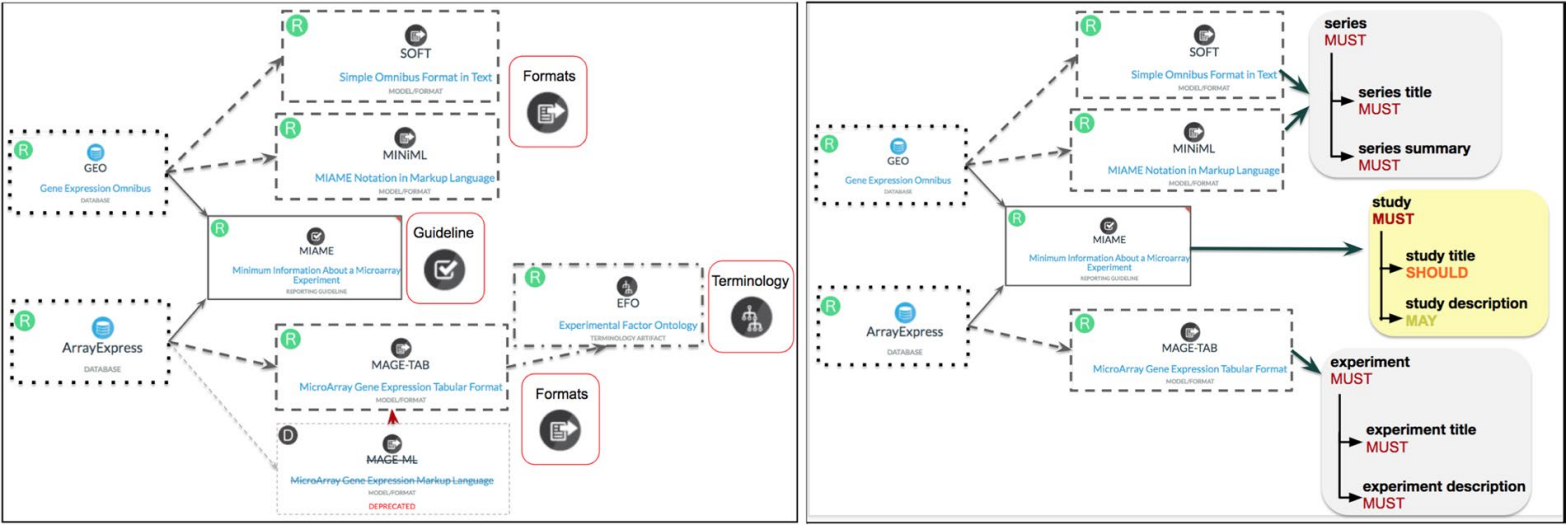
Difference in representation of the MIAME checklist in two public repositories: GEO and ArrayExpress. (**A**) GEO (10.25504/FAIRsharing.5hc8vt) and ArrayExpress (10.25504/FAIRsharing.6k0kwd) are two databases highly recommended by journals and funders data policies, and both implement the community-defined MIAME reporting guideline to describe microarray experiment (10.25504/FAIRsharing.32b10v), among others. The implementation of MIAME is done via several formats (used to upload and download datasets from these two databases), which include SOFT (10.25504/FAIRsharing.3gxr9) and MINiML (10.25504/FAIRsharing.gaegy8) for GEO; MAGE-ML (10.25504/FAIRsharing.x964fb) that is now deprecated and superseded by MAGE-TAB (10.25504/FAIRsharing.ak8p5g) for the ArrayExpress, which also uses the EFO terminology (10.25504/FAIRsharing.1gr4tz) to annotate the metadata. **(B)** Using a few metadata requirements from MIAME as example (namely: study, study title, study description) we illustrate how the metadata labels, along with their level of requirement (must, should, may), varies across the formats used by the two databases.

Since the early 2000s, a substantial number of community-based standardisation initiatives have worked to harmonise the reporting and sharing of the metadata of datasets, and more recently, of software code and other digital objects. In the Life Sciences, over a thousand metadata standards have been created and/or implemented by several thousand data repositories, as inventoried by FAIRsharing (https://fairsharing.org/standards). These community-driven efforts encompass: minimum information checklists or reporting guidelines; terminology artefacts or semantics (ranging from dictionaries to ontologies); models and formats or syntax. Reporting guidelines play a pivotal role, because they define the key descriptors the community sees as the necessary and sufficient information that must be reported to contextualize and understand datasets. These reporting guidelines, however, are usually intended for human consumption and are, in their majority, in narrative form and therefore prone to interpretation ambiguities, making automatic validation and compliance against metadata standards a difficult and approximate task (see Fig. 1). The reporting guidelines serve as an initial phase that facilitates the development of models and formats, defining the formal structures and relationships of information to be reported along with transmission formats to facilitate data exchange. These models and formats often mandate the use of one or more terminologies, which provide definitions and univocal identification for concepts or to define value-sets.

The uptake of these metadata standards by the research community, data repositories, tools developers, services providers, and policy makers has been slow and uneven, mainly because of a lack of incentives and information, but also due to perceived or real technical challenges. However, the community mobilization around FAIR-enabling tools and services, and the network effect of resources like FAIRsharing^8^, are bringing a renewed energy and attention to these issues. The question is therefore how to develop tools to support changes to the maturity level of reporting guidelines, moving away from narratives to formal representations and promoting reuse.

### The need for modular, reusable, metadata models

The need for composability and reusable metadata elements is not new and has been the focus of much academic research^9,10^. The notion of Common Data Elements (CDE), the ISO11179 model for metadata registry^11^ and related tools, such as caCORE and caDSR^12^, are examples of existing solutions to those problems^13^. However, for grassroots efforts, such as the former MIBBI^14^ communities now part of FAIRsharing the CDE models are often too complex, the ISO specifications are behind paywall, and the availability of maintained open source software is limited, and combined, these represent significant hurdles^15,16^. The need for the reuse of machine-actionable metadata was also the focus of Metadata for Machine^17^, and a series of workshops organized by the GO-FAIR initiative (https://www.go-fair.org), which we contributed to bringing in our experience with the ISA community since 2010^18^.

In the ISA metadata authoring tool, the reporting guidelines were translated into XML-based templates, building on ISA syntactic patterns that take into consideration the type of sample, the experimental condition, the technology employed and so on. In the last decade, however, web technologies, in particular JavaScript Object Notation (JSON) Schema (https://json-schema.org) and schema and JSON-LD (http://json-ld.org), have gained huge popularity, owing to the relative ease of use and integration with javascript based components, even displacing well entrenched technology, such as XML, owing to the relative ease of use and integration with javascript based components. For example, and have been adopted by major bioinformatics projects such as GA4GH^19^, Human Cell Atlas^20,21^, Biolink^22^ projects and its reliance on LinkML^23^ for providing schema driven metadata. In response to this change, and end user requests, we pivoted to use this stack, and this manuscript presents our progress in this area.

## Results

We focused our efforts on a selected subset of reporting guidelines in Life Science, described in Table 1. We worked to improve the process, researching new methods to create standards-derived metadata elements that are machine-actionable, modular, and reusable for composition in intelligent authoring templates, tools, validation and assessment tools at scale. We build on the notion of ‘atomic’ metadata elements, and an architecture that encourages reuse and modularity based on JavaScript Object Notation (JSON) technologies and a set of open source tools, which can be used to explore, create, extend and validate these metadata models. We apply the method to a set of reporting guidelines to illustrate how to create a machine-readable reporting guideline *de novo*, and how to merge two existing guidelines into a new set of schemas.

**Table 1:** The set of reporting guidelines we selected to illustrate our approach.

Reporting guideline name	Domain coverage	Format	Date of creation	FAIRsharing record DOI
MIAME	Transcriptomics	Textual, PDF file	1999	10.25504/FAIRsharing.32b10v
MIACA	Cellular assay	XSD	2006	10.25504/FAIRsharing.7d0yv9
MIFlowcyt	Flow cytometry	Textual, PDF file	2007	10.25504/FAIRsharing.kcnjj2
MINSEQE	High-throughput nucleotide sequencing	Textual, PDF file	2008	10.25504/FAIRsharing.a55z32
MIXS-MIMARKS	Nucleotide sequencing from environmental samples	Excel, XML file	2011	10.25504/FAIRsharing.zvrep1
MIACME	Cell migration assay	generate *ab initio as JSON Schema*	2016	10.25504/FAIRsharing.vh2ye1
MIAPPE	Plant phenotyping	Excel, TSV file	2018	10.25504/FAIRsharing.nd9ce9

These encompassed examples in narrative and formalized format, older and newer work, and include some that have a domain overlap in order to test the composability capability.

### Building machine-actionable metadata models

#### Target users, use cases and technology

Our work aims to assist developers of data annotation standards, data managers and data stewards to achieve community requirements compliance and FAIR assessments. Two main use cases (UC) have guided our work:

- UC1: move the maturity level of reporting guidelines away from the narrative form to machine actionability and formal representation.
- UC2: how to combine two existing guidelines into a new set of schemas.
- This second use case can assist the first one and addresses the need to ensure compatibility, detect overlaps and deal with redundant requirements, while retaining unique metadata descriptors.

JSON was selected to express metadata requirements from existing *but not machine-actionable* reporting guidelines, for it is the *de facto* standard for developing web-oriented components and services. Specifically, we used JSON-Schema and JSON-LD technologies, in order to decouple the annotation requirements from a domain model. JSON Schema is a vocabulary to validate JSON files, and JSON-LD is an extension of JSON to support linked data: a way to create a machine-readable and standard way to share data on the web respectively.

A dedicated GitHub repository was set up to act as a catalogue of Minimum Information Requirement schemas (https://github.com/FAIRsharing/mircat). The MIRcat GitHub repository hosts JSON Schema representations and associated JSON-LD context files for the efforts listed in Table 1. Reporting requirement validation can be done by software agents either against the JSON Schemas or the JSON instance documents. To demonstrate the approach, we used FlowRepository (http://flowrepository.org/), an archive of Flow Cytometry data, as a test bed. Using the MIflowCyt specification documentation, we created a set of JSON Schemas (https://github.com/FAIRsharing/mircat/tree/master/miflowcyt). Retrieving experiments metadata, XML instances were transformed to JSON and the linked data attributes were injected to obtain the final JSON-LD instances that were then validated against the JSON Schema set. The aim of this work was two-fold: first, to show the feasibility of the JSON schema based approach, and second, to provide a baseline for follow-up work, demonstrating the ability to perform validation of instance documents and JSON-LD based semantic injection.

#### Formal expression and annotation of MI checklists

Since many checklists are only available in textual forms and lack univocal formal representations, their use by both humans and machines in a consistent manner is actually seriously limited. Therefore, a key task consists in formalizing textual checklists into machine-actionable representations. This formalization step ideally seeks reusability and composability, something that implies checklists decomposition to their simplest entities. We chose to decompose the selected checklists by extracting their common and reusable entities in dedicated files, and relying on JSON-Schema specification based models. This step allows authors to unambiguously specify which entities, relationships, attributes and properties are represented in a model, such that different communities or agents can reuse, fully or partially, an existing model.

The provision of explicit semantics requires annotating each entity and field with ontology terms. This can be achieved by creating JSON-LD context files with the support of the JSON-ScheeLD toolkit. The decoupling of the semantic layer, provided by the JSON-LD context files, and syntactic layers (the JSON schemas) allows different communities to customise their ontology sources without disrupting the underlying model. This therefore presents a greater chance of reuse of schema components (JSON schema), and in turns, possibly reducing the interoperation friction inherent to these processes^24^.

#### Annotate, explore and validate

**Fig. 2:**
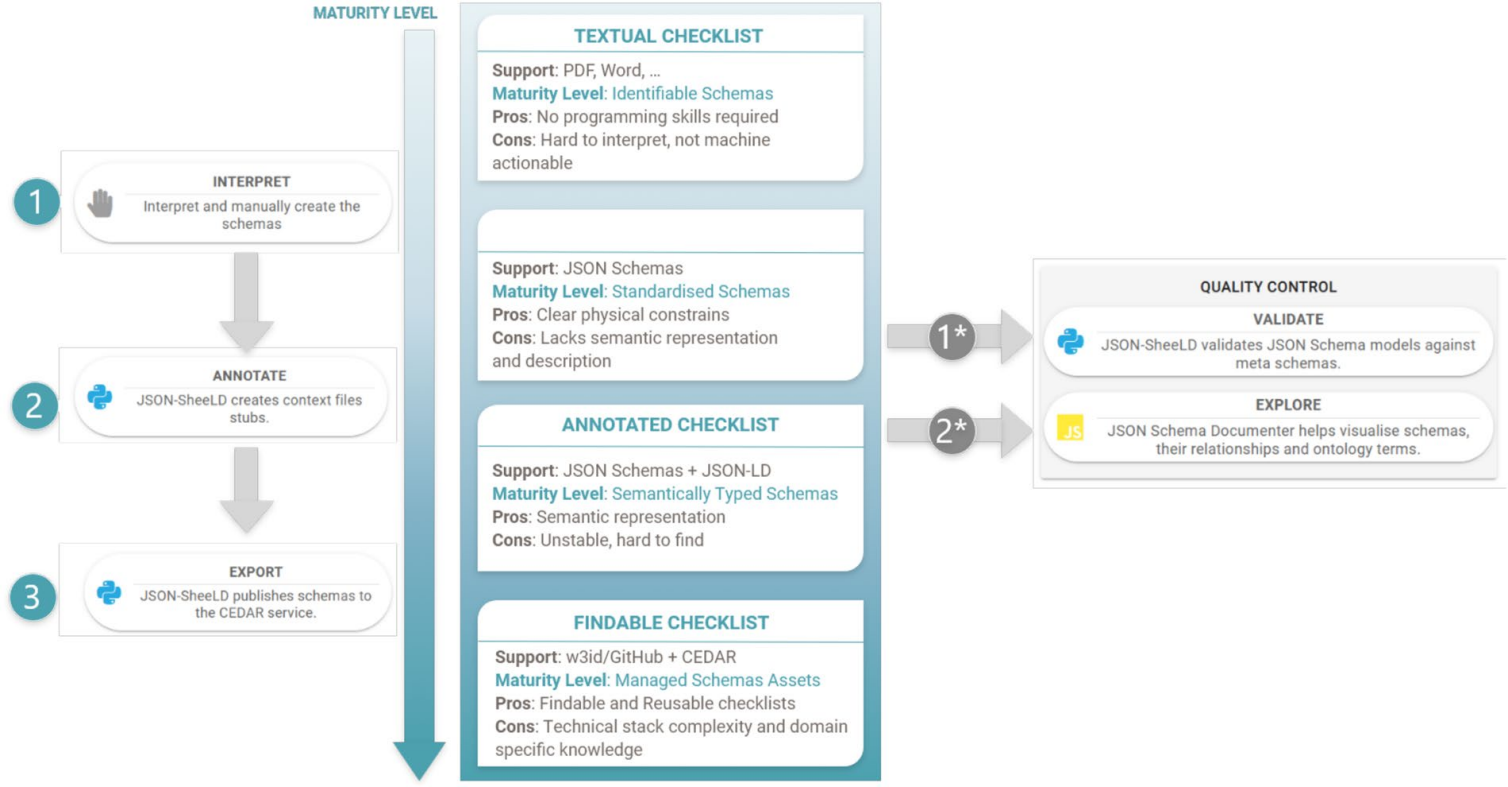
How to create a reporting guideline that is machine-readable *ab initio*. (1) A checklist/reporting guideline is formally expressed as JSON schemas. 1*) Quality Control step: *JSON ScheeLD* provides the means to validate the model against the JSON Schema specification; and the *JSON Schema Documenter* helps visualise models in the browser. (2) *JSON ScheeLD* creates JSON-LD context file stubs and user provides the mapping manually. 2*) Quality Control step: use *JSON Schema Documenter* to verify that all the fields are mapped to an ontology term. (3) Export to the CEDAR API and provide stable identifiers.

The production of JSON Schemas often lacks in terms of visualisation support, hampering interactions between end-users and developers. A number of rendering tools exist but they have either limited capabilities (e.g. failure to resolve complex schemas or to account for context files) or are tied to large frameworks (e.g. cloudflare, https://www.cloudflare.com). To help users visualize JSON Schema based models, we created the JSON Schema *Documenter*, a lightweight, AngularJS-based client-side web application which presents, *in-situ*, the associated semantic markup extracted from the JSON-LD context files. JSON-ScheeLD tool facilitates the binding of schema elements to semantic terms by creating JSON-LD ontology-specific context file stubs and the visualization of complex sets of JSON Schemas. The process of mapping to ontologies remains a manual task that the tool cannot automate at the moment. Figure 2 illustrates the process required to create a new reporting guideline in a machine-readable and annotated form.

#### Compare and merge

The technology used to formally express the reporting guidelines relies on the separation between the syntactic layer (the JSON schema specifications in our tool) and the semantic layer (associating json types and properties to ontology terms via JSON-LD specifications). We provide the ability to compare sets of schemas, based either on syntactic or semantic comparisons, and it is down to the user to select which function to invoke. The syntactic comparison relies on the *DeepDiff* python library and returns the differences between two given objects. The semantic comparison relies on our custom *SemDiff* comparator, which consists in a uri matching function, assembled from the prefixes and identifiers found in the JSON-LD context files. It could be extended in the future to leverage more sophisticated ontology mapping tools such as, for example, LogMap^25,26^.

**Fig. 3:**
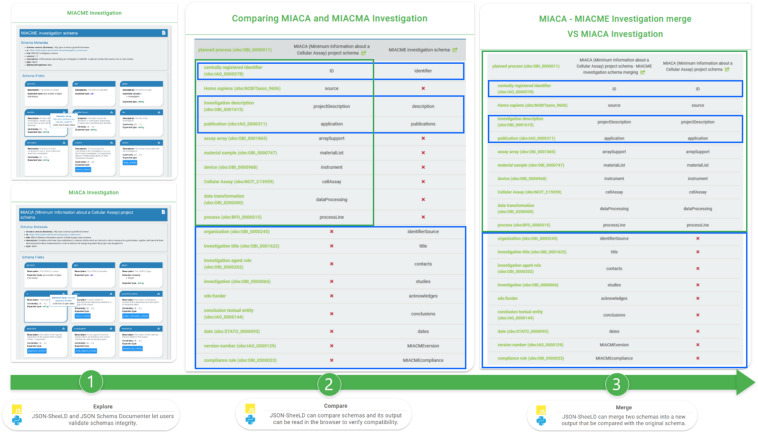
How to merge two existing guidelines into a new set of schemas. (1) A developer uses the *JSON Schema Documenter* to explore the different guidelines, MIACME and MIACA. (2) *JSON ScheeLD* relies on the context files to compare the two given models and outputs a file readable by the *JSON Compare Viewer*. This allows the developer to see which fields are semantically identical. (3) *JSON ScheeLD* pulls the fields from the MIACME model and injects them into the MIACA if they are missing and creates a whole new set of schemas and context files. Directionality is important: merging MIACME into MIACA will not produce the same result as merging MIACA into MIACME. (4) After the merge is complete, the developer can go back to step 2 and compare the new model with the old one to ensure quality control.

The output of the semantic comparison is rendered by the JSON *Compare Viewer* front-end app (https://github.com/FAIRsharing/JSONschema-compare-and-view). The same output may also be used by the JSON ScheeLD *merge* function to generate a new set of schemas, as illustrated with Fig. 3, where MIACME is merged into the MIACA schema. During this process, the provenance of the metadata descriptors from the MIACA reporting guideline was preserved.

#### Towards CEDAR compatible schemas

Production grade schemas are hosted on a dedicated GitHub repository (https://github.com/FAIRsharing/mircat), licensed under BSD3, and issued a stable resolvable identifier by relying on the w3id service. In addition, as part of our collaboration with the Center for Expanded Data Annotation and Retrieval (CEDAR)^27^, we provided a conversion mechanism to allow the reporting guidelines to be deposited to the CEDAR Workbench (https://jsonldschema.readthedocs.io/en/latest/cedar/cedarIndex.html). It should be noted that to convert to the CEDAR format, a pre-processing of the native JSON Schemas is necessary and is included in the JSON ScheeLD component. With the set of functions mentioned in this section, minimal Information checklists can be represented in readable, accessible, interoperable and reusable form. Table 2 lists the key functions present in JSON ScheeLD and relates them to the FAIR dimensions.

## Discussion

The core of our work consisted in expressing reporting guidelines formally, i.e. moving away from free text descriptions, to machine actionable formats, essentially increasing the data maturity level of the guidelines. This step is time consuming, hard to automate, and it is a manual process that involves domain experts. In the following sections, we discuss the four main challenges, which are also areas of future work, and how to mitigate them: (i) the consistency of the semantic markup, (ii) the versioning of the reporting guidelines, (iii) the ambiguities of JSON Schema, and (iv) the difficulties with comparing at syntactic and semantic level.

Upon completing the formalization, the next step consisted in performing a semantic markup on the JSON Schema attributes. The choice of the semantic resources is dictated by various constraints, and it is important to clearly articulate the selection process of these resources and to document the process for term selection. A simple selection based on ‘string match’ is insufficient owing to the homonymy issue. For instance, the notion of ‘library’ in the MINSEQE reporting guideline, which is focused on nucleic acid sequencing, differs significantly from the one in the MIACA reporting guideline, where ‘library’ refers to a collection of perturbation agents such as silencing RNA clones. Therefore, beyond the term label, it is essential to consider key metadata of an ontology class^28^, such as definition, synonyms and examples. The semantic markup is particularly important as the *JSON ScheeLD Compare* feature uses the compact URIs found in the JSON-LD context file to match elements and decide whether to merge attributes or not. It should be noted that the *JSON ScheeLD Merge* feature does not evaluate ontology classes based on more advanced heuristics, such as calculating semantic similarity distances.

Further enhancements are needed to refine the expression of the constraints under JSON Schema. Since the draft 3.0 version, JSON Schema specifications have keywords to allow schema referencing and reuse, affording the creation of more complex networks of schemas. However, the process of injecting semantic types into JSON instance files using JSON-LD context files in a programmatic and dynamic way faces hard limitations. One such case is the difficulty to inject the correct @type attribute during the JSON-LD conversion when JSON schema properties rely on the ‘anyOf’ and ‘oneOf’ keywords, which point to a range of possible types. The injection requires validating every object against each of the specified schema to know which type(s) to inject. If an object validates against multiple types but only one type is allowed at a time, the algorithm cannot decide which one to inject. Moreover, the ability to enforce multiple types with the ‘allOf’ keyword can cause the provision of contradictory constraints, preventing any further validation. This is the case when an object should validate against two or multiple schemas that provide different constraints on the same property, for instance if one schema describes an identifier as a number whereas the other describes it as a string. Unfortunately, the JSON Schema specification does not mention the existence of such cases, therefore the validators do not account for such cases and will not catch these contractions. As result, it has been our responsibility to produce unambiguous JSON Schema documents.

When working with community-defined reporting guidelines, one needs to prepare for change as guidelines can evolve. Should this happen, the related schemas would need to be updated and changes may break pre-existing compliance. To mitigate this issue, a versioning mechanism should be put in place; all versions should remain accessible through a stable and persistent URL, also to avoid breaking objects depending on these schemas. Therefore, schemas themselves need to be FAIR and we looked into organizing the various reporting requirement release versions under the MIRcat repository and around w3id folders holding the different releases. As part of future work, we plan to ensure that the JSON-ScheeLD tool is able to deal with multiple versions of a reporting requirement.

Beside the technical challenges described above, our prototype work demonstrates that the goals of machine-actionable guidelines and compositional metadata elements are achievable. We encourage feedback and contributions from developers interested to explore the tools, the schemas and the compositional metadata elements produced to date through our GitHub code repository. While most machine-readable guidelines can be constructed with a high level of confidence and precisely, some would require vetting by the original developers of the reporting guidelines. To facilitate this, we plan to link the community-defined reporting guidelines found in FAIRsharing with their respective machine-actionable models stored in the MIRcat repository.

In the long run, an ideal scenario is one where reporting guidelines are no longer developed as unstructured narrative, but are expressed with sufficient level of formalization to enable validation by machines from a set of available components. The long-term goal is to develop a consistent and coordinated approach to machine-readable profiles. Formally-defined reporting requirements, with semantic markup, cardinality information, and value-set definitions can also be used to bootstrap the creation of project-specific metadata profiles, possibly with the assistance of software agents powered by models trained on these.

We will also continue to foster a community-wide discussion via our participation in international initiatives on metadata authoring and FAIR assessment including the Metadata for Machine workshops (by GO-FAIR, and where CEDAR and FAIRsharing are also part of), and communities activities in international data infrastructures such as ELIXIR (https://elixir-europe.org). The need for coordination among community standards and their interoperability are not trivial issues^29^. There are social and technical challenges. Nevertheless, it is crucial to foster convergence, modularity and interoperability among the community-standards if we are to realise the vision of FAIR data.

## Methods

To demonstrate the feasibility of the approach, we used the subset of reporting guidelines described in Table 1. The following criteria were used for the selection: 1) at least one narrative reporting guideline (to assess the extraction from textual representation), 2) at least one formalized reporting guideline (to assess the extraction from formal representation) and 3) a domain overlap in at least one component (to test composability capability). For the latter criteria, we included MIACA and MIACME, which cover cellular systems, and MINSEQE and MIXS-, MIMARKS, which cover nucleic acid sequencing.

The reporting guidelines all differ in terms of scope as well as development and maturity stages. They also range from purely narrative artefacts (with possible ambiguous definitions) to fully formalized models, which are supported by a model-based representation either in the form of a UML diagram, XML schema, JSON schema or Resource Description Framework (RDF) representation. For the reporting guidelines with a formal representation, an interpretation and formalisation step was performed independently by two domain experts, and reconciled by a third expert. The task consisted in identifying key concepts (as defined by the authors of the reporting guideline) and building an entity/relationship model from the textual definition^30^. Finally, for each of the identified entities, a concept identifier from a selected ontology was linked to it.

The process, referred to as metadata atomization and markup, relies on the identification and consistent use of semantic resources. We selected schema.org (https://schema.org) and OBO Foundry^31^ resources as they provide two distinct and complementary functions. The former is orientated towards data discovery and findability, and the latter focuses on the coherent representations of the biological domains, which are under-represented in schema.org. The metadata atomization and markup is primarily a manual process and hard to automate. To select suitable ontology terms for the annotation of the schemas types and fields, we relied on Google Spreadsheet and on Ontomaton, a Google Spreadsheet plugin^32^ part of the ISA software suite, which allows querying ontologies by accessing the NCBO Bioportal^33^, the EBI Ontology Lookup Service^34^ and the LOD vocabulary service^35^. The NCBO Annotator^36^ service was also used in the first round of annotation, after having identified a subset of resources to query. All terms suggestions were reviewed prior to approval.

After having reviewed an earlier attempt to represent reporting guidelines with a purely RDF approach (MIM vocabulary)^37^, our choice to use JSON Schema and JSON-LD was guided by three main observations. First, JSON Schema and JSON are extremely popular formats for developing web-oriented components. Second, a number of large-scale biology related programs have adopted a data modelling approach rooted in JSON Schema technology. For instance, the Human Cell Atlas project^20^ is developing metadata models relying on JSON Schema draft 7.0 (https://github.com/HumanCellAtlas/metadata-schema). Third, JSON Schema and JSON documents are widely supported, with dozens of libraries available for reading, writing, validating, parsing and rendering the information. While there is support to validate RDF graphs against a set of conditions (e.g., SHACL http://datashapes.org/forms.html, and ShEx shape expressions https://www.w3.org/2015/03/ShExValidata, https://github.com/CSIRO-enviro-informatics/shacl-form), these technologies were less pervasive when the project was started Table 2.

**Table 2:** (*) The CEDAR and FlowRepository API require API keys.

FAIR Dimension	Output characteristics	JSON ScheeLD function
Findable	• Schemas are identified by W3id identifiers; • Schemas are exportable via the CEDAR API*.	**JSON to CEDAR Conversion**: https://jsonldschema.readthedocs.io/en/latest/cedar/cedarUsage.html
Accessible	• Schemas are retrievable via https GET method; • Software is API ready.	**Manual**: GitHub hosting and w3id redirection **API**: https://jsonldschema.readthedocs.io/en/latest/API/apiUsage.html
Interoperable	• Schemas are available as JSON with associated JSON context files; • Schemas and instances are validated; • Provides an example of XML to JSON-LD instance conversion using MiFlowCyt data*; • Supports multiple ontologies to describe the same resources.	**Context files helpers**: https://jsonldschema.readthedocs.io/en/latest/utils/schemaUtilities.html **Validation**: https://jsonldschema.readthedocs.io/en/latest/validation/validationUtilities.html **XML to JSON-LD Conversion**: https://jsonldschema.readthedocs.io/en/latest/validation/validationUsage.html
Reusable	• Schemas and softwares are available under licensing BSD-3; • Schemas support the declaration of data licences. • Schemas provenance information are available with PROV from CEDAR; • Schemas can be compared and merged.	**Licence:** https://github.com/FAIRsharing/jsonldschema/blob/master/LICENSE.md **Provenance:** https://jsonldschema.readthedocs.io/en/latest/cedar/cedarUsage.html?highlight=provenance **Merge:** https://jsonldschema.readthedocs.io/en/latest/semDiff/merger.html

To know more, refer to the following documentations: https://metadatacenter.github.io/cedar-manual/advanced_topics/b2_cedars_api/ and https://flowrepository.org/images/pdf/FlowRepositoryAPI.pdf.

For the implementation, we followed software engineering best practices, and GitHub^38^ was used to archive, version, release and document the code. Object oriented approach was used and Agile methodology applied, along with pair programming and systematic code review through a branching and pull request approach. Code quality was ensured through unit testing, as well integration testing. The GitHub repositories were set up to enable continuous integration using Travis CI (https://travis-ci.com/auth) and Coveralls (https://coveralls.io/) hooks, allowing building of the infrastructure on each commit and notification of the developers systematically on any critical failure. The documentation is available as readthedocs (https://jsonldschema.readthedocs.io/). It covers the entire set of components making up FAIRsharing JSON-LD Schema library, namely: (i) the JSON-LD Schema python module, which provides the functionality to deal with JSON Schemas accompanied by JSON-LD context files, allowing JSON Schemas for JSON-LD instances, (ii) the AngularJS web application JSON Schema *Documenter*, and (iii) the AngularJS web application JSON Schema *Compare and View* that supports visualisation of the comparison.

## Data Availability

The data generated during the current work are available as FAIR and machine actionable reporting guidelines from the FAIRsharing/mircat GitHub repository from the following link: https://github.com/FAIRsharing/mircat. The latest release is available from Zenodo^39^.

## Code availability

All the code produced for the present study is available from the following GitHub repositories:

- http://github.com/fairsharing/jsonldschema

- https://github.com/fairsharing/jsonschema-documenter

- https://github.com/FAIRsharing/JSONschema-compare-and-view

Supporting documentation is available from https://jsonldschema.readthedocs.io.
